# Time Table

**Published:** 1857-11

**Authors:** 


### TIME TABLE.

THE following useful table is taken from *Dinsmore's Railroad Guide.* When it is noon, or twelve o'clock, at Washington City, it is fourteen minutes past twelve at Albany, New York. Places *west* of Washington have slower, while those east of Washington have earlier time than at Washington.

| NOON AT WASHINGTON, D. C. | | | |
|---|---|---|---|
| Albany, N. Y. | 12 | 14 | P.M. |
| Augusta, Ga. | 11 | 41 | A.M. |
| Augusta, Me. | 11 | 31 | " |
| Baltimore, Md. | 12 | 02 | P.M. |
| Beaufort, S. C. | 11 | 47 | A.M. |
| Boston, Mass. | 12 | 24 | P.M. |
| Bridgeport, Ct. | 12 | 16 | " |
| Buffalo, N. Y. | 11 | 53 | A.M. |
| Burlington, N. J. | 12 | 09 | P.M. |
| Burlington, Vt. | 12 | 16 | " |
| Canandaigua, N. Y. | 11 | 59 | A.M. |
| Charleston, S. C. | 11 | 49 | " |
| Chicago, Ill. | 11 | 18 | " |
| Cincinnati, O. | 11 | 31 | " |
| Columbia, S. C. | 11 | 44 | " |
| Columbus, O. | 11 | 36 | " |
| Concord, N. H. | 12 | 23 | P.M. |
| Dayton, O. | 11 | 32 | A.M |
| Detroit, Mich. | 11 | 36 | " |
| Dover, Del. | 12 | 06 | P.M. |
| Dover, N. H. | 12 | 37 | " |
| Eastport, Me. | 12 | 41 | " |
| Frankfort, Ky. | 11 | 30 | A M. |
| Frederick, Md. | 11 | 59 | " |
| Fredericksburg, Va. | 11 | 58 | " |
| Frederickton, N. B. | 12 | 42 | P.M. |
| Galveston, Texas | 10 | 49 | A.M. |
| Gloucester, Mass. | 12 | 26 | P.M. |
| Greenfield, " | 12 | 18 | " |
| Hagerstown, Md. | 11 | 58 | " |
| Halifax, N. S. | 12 | 54 | P M. |
| Harrisburg, Pa. | 12 | 01 | " |
| Hartford, Ct. | 12 | 18 | " |
| Huntsville, Ala. | 11 | 21 | A.M. |
| Indianapolis, Ind. | 11 | 26 | " |
| Jackson, Miss. | 11 | 08 | " |
| Jefferson, Mo. | 11 | 00 | " |
| Kingston, Can. | 12 | 02 | P.M. |
| Knoxville, Tenn. | 11 | 33 | A.M. |
| Lancaster, Pa. | 12 | 03 | P.M. |
| Lexington, Ky. | 11 | 31 | A.M. |
| Little Rock, Ark. | 11 | 00 | " |
| Louisville, Ky. | 11 | 26 | " |
| Lowell, Mass. | 12 | 23 | P.M. |
| Lynchburg, Va. | 11 | 51 | A M. |
| Middletown, Ct. | 12 | 18 | P.M. |
| Milledgeville, Ga. | 11 | 35 | A.M. |
| Milwaukee, Wis. | 11 | 17 | " |
| Mobile, Ala. | 11 | 16 | " |
| Montpelier, Vt. | 12 | 18 | P.M. |
| Montreal, Can. | 12 | 14 | " |

| NOON AT WASHINGTON, D. C. | | | |
|---|---|---|---|
| Nashville, Tenn. | 11 | 21 | A.M. |
| Natchez, Miss. | 11 | 03 | " |
| Newark, N. J. | 12 | 11 | P.M. |
| New Bedford, Mass. | 12 | 25 | " |
| Newburg, N. Y. | 12 | 12 | " |
| Newburyport, Mass. | 12 | 25 | " |
| Newcastle, Del. | 12 | 06 | " |
| New Haven, Ct. | 12 | 17 | " |
| New London, Ct. | 12 | 20 | " |
| New Orleans, La. | 11 | 08 | A.M. |
| Newport, R. I. | 12 | 23 | P.M. |
| New York, N. Y. | 12 | 12 | " |
| Norfolk, Va. | 12 | 03 | " |
| Northampton, Mass. | 12 | 18 | " |
| Norwich, Ct. | 12 | 20 | " |
| Pensacola, Flor. | 11 | 20 | A.M. |
| Petersburg, Va. | 11 | 59 | " |
| Philadelphia, Pa. | 12 | 08 | P.M. |
| Pittsburg, Pa. | 11 | 48 | A.M. |
| Plattsburg, N. Y. | 12 | 15 | P.M. |
| Portland, Me. | 12 | 28 | " |
| Portsmouth, N. H. | 12 | 25 | " |
| Prairie du Chien, Wis. | 11 | 04 | A.M. |
| Providence, R. I. | 12 | 23 | P.M. |
| Quebec, Can. | 12 | 23 | " |
| Racine, Wis. | 11 | 18 | A.M. |
| Raleigh, N. C. | 11 | 53 | " |
| Richmond, Va. | 11 | 58 | " |
| Rochester, N. Y. | 11 | 57 | " |
| Sacket's Harbor, N. Y. | 12 | 05 | P.M. |
| St. Anthony Falls | 10 | 56 | A.M |
| St. Augustine, Flor. | 11 | 42 | " |
| St. Louis, Mo. | 11 | 07 | " |
| St. Paul, Min. | 10 | 56 | " |
| Sacramento, Cal. | 9 | 02 | " |
| Salem, Mass. | 12 | 26 | P.M. |
| Savannah, Ga. | 11 | 44 | A.M. |
| Springfield, Mass. | 12 | 18 | P.M. |
| Tallahassee, Flor. | 11 | 30 | A.M. |
| Toronto, Can. | 11 | 51 | " |
| Trenton, N. J. | 12 | 10 | P.M. |
| Troy, N. Y. | 12 | 14 | " |
| Tuscaloosa, Ala. | 11 | 18 | A.M. |
| Utica, N. Y. | 12 | 08 | P M. |
| Vandalia, Ill. | 11 | 18 | A.M. |
| Vincennes, Ind. | 11 | 19 | " |
| Wheeling, Va. | 11 | 45 | " |
| Wilmington, Del. | 12 | 06 | P.M. |
| Wilmington, N. C. | 11 | 56 | A.M. |
| Worcester, Mass. | 12 | 21 | P.M. |
| York, Pa. | 12 | 02 | " |

